# Potential Impact of a 2-Person Security Rule on BioSafety Level 4 Laboratory Workers

**DOI:** 10.3201/eid1507.081523

**Published:** 2009-07

**Authors:** James W. LeDuc, Kevin Anderson, Marshall E. Bloom, Ricardo Carrion, Heinz Feldmann, J. Patrick Fitch, Joan B. Geisbert, Thomas W. Geisbert, Michael R. Holbrook, Peter B. Jahrling, Thomas G. Ksiazek, Jean Patterson, Pierre E. Rollin

**Affiliations:** Author affiliations: University of Texas Medical Branch, Galveston, Texas, USA (J.W. LeDuc, M.R. Holbrook, T.G. Ksiazek); National Biodefense Analysis and Countermeasures Center, Frederick, Maryland, USA (K. Anderson, J.P. Fitch); National Institutes of Health, Hamilton, Montana, USA (M.E. Bloom, H. Feldmann); Southwest Foundation for Biomedical Research, San Antonio, Texas, USA (R. Carrion Jr, J. Patterson); Boston University School of Medicine, Boston, Massachusetts, USA (J.B. Geisbert, T.W. Geisbert); National Institutes of Health, Bethesda, Maryland, USA (P.B. Jahrling); and Centers for Disease Control and Prevention, Atlanta, Georgia, USA (P. Rollin)

## Abstract

Directors of all major BioSafety Level 4 (BSL-4) laboratories in the United States met in 2008 to review the current status of biocontainment laboratory operations and to discuss the potential impact of a proposed 2-person security rule on maximum-containment laboratory operations. Special attention was paid to the value and risks that would result from a requirement that 2 persons be physically present in the laboratory at all times. A consensus emerged indicating that a video monitoring system represents a more efficient, economical standard; provides greater assurance that pathogens are properly manipulated; and offers an increased margin of employee safety and institutional security. The 2-person security rule (1 to work and 1 to observe) may decrease compliance with dual responsibilities of safety and security by placing undue pressure on the person being observed to quickly finish the work, and by placing the observer in the containment environment unnecessarily.

## Abstract 2

DOI: 10.3201/eid1507.081523

Biosafety Level 4 (BSL-4) maximum-containment laboratories are designed to provide a safe environment for the study of some of the world's most serious infectious diseases, including several that are of grave concern as potential bioterrorism threats. Given longstanding concerns for the safety of persons working in maximum-biocontainment laboratories, systems and procedures have been identified for these facilities to identify safety risks and initiate appropriate action in the case of an accident or potential exposure (*1*). Consequently, these facilities have had a distinguished safety record for >30 years; no clinical infections, no major incidents in operation of the physical facilities, and no escape of any agent with clinical consequences for neighboring communities have occurred (*2**,**3*)

Rigorous requirements are already in place and fully outlined in the standard operating procedures of all BSL-4 laboratories covering the safety and security procedures to be followed when removing infectious material from the facility. These requirements are carefully reviewed by external experts as part of the select agent oversight and approval process and strictly enforced by leadership of all BSL-4 laboratories. After recent accusations that a laboratory worker may have been associated with the 2001 anthrax attacks in the United States, additional concerns regarding surety or security in BSL-4 laboratories have been raised (*4*). Such concerns acknowledge the need to establish policies and programs to ensure that BSL-4 laboratory workers do not engage in malfeasance that could lead to an intentional release of infectious agents.

Directors of all major BSL-4 laboratories in the United States met in 2008 to review the current status of biocontainment laboratory operations and to discuss options for comprehensive risk assessment and management approaches to ensure a working environment in which safety and security are paramount. It is critical in the process of developing new standards that measures to enhance security do not inadvertently increase safety risks for persons conducting biocontainment laboratory research.

Various approaches to strengthen the safety and security of maximum-containment laboratory workers and their environment have been proposed and were considered (*5*). These included 1) having 2 persons physically present within the BSL-4 facility any time that work is being performed (the 2-person rule); 2) use of surveillance cameras to monitor workers in the BSL-4 facility; and 3) use of radios or other means of communication between workers inside the laboratory and qualified contact persons outside the actual BSL-4 environment.

Typically, the daily flow of work in a biocontainment laboratory involves >2 persons working near each other. Although the primary role of each person is to perform his or her work and not to monitor their coworker, there would still be ample opportunity to render aid if needed or to observe untoward activities. The risk issues of the 2-person rule arise when the normal activity in the laboratory is insufficient to satisfy the rule in every active area of the BSL-4 laboratory. These issues will occur frequently outside normal working hours, i.e., evenings, weekends, or holidays, and also during regular working shifts as project-specific tasks are completed asynchronously. The key implementation issue for a 2-person rule is when staff members are required to enter or extend time in containment solely to fulfill the security requirement. This personnel-centric approach has some serious shortcomings and in many circumstances may increase the safety risk for laboratory personnel (*6*). Effectively, the presence of an observer for the sole purpose of achieving a 2-person requirement would contribute to perceived time pressures, stress, distractions, and interruptions, all of which are factors identified by human performance management as error precursors (*7*). Independent assessments of such regulations, as they pertain to select agents and biological research, have reached the same conclusion (*8*).

BSL-4 directors recognized that some activities carry substantial inherent risk. Thus, there is value in requiring >2 persons to be present inside the BSL- facility for personal safety. These risks include but are not limited to 1) manipulations of animals, particularly nonhuman primates; 2) maintenance work that would require climbing on ladders and other situations where falls might occur; 3) movement of heavy or cumbersome objects; and 4) supervision and training of staff members who have not yet qualified for independent access.

Surveillance video monitoring and data storage are existing elements of an enhanced safety and security program (*9**,**10*). All BSL-4 laboratories today are equipped with remote-controlled surveillance cameras that can track a person within the maximum-containment laboratory. Cameras can be programmed to record continuously or upon activation by a motion detector or access control device in specific rooms or on freezers; observers outside the BSL-4 laboratory can monitor work being conducted, readily determine if an emergency or security breach has occurred, and initiate the proper emergency response sequence if necessary. Specific procedures and capabilities vary from institution to institution, but in all instances cameras are monitored by appropriate security and program staff who are well versed in the institutional security and emergency response plans for BSL-4 operations. Typically, images can be securely stored for an indefinite period of time. Furthermore, freezers containing select agents and other high-risk pathogens are usually provided with an access system that records the identity of a person taking material from the repository. When coupled with camera surveillance, the system enables an accurate, complete, and potentially permanent record of persons gaining access to pathogens. These powerful tools are already in place and are used to monitor work undertaken in the laboratories.

All BSL-4 laboratories have telephones, often equipped with flashing alerts for incoming calls. Also, most BSL-4 facilities in the United States have some form of radio communication available to persons working in the suite, although use of radios has not been universally adopted because the technology has not yet been tailored to this unique environment. Wall-mounted video screens capable of displaying alerts are now available in most BSL-4 laboratories, as are panic buttons that can be used in emergency situations. These modes of communication between persons working within the BSL-4 laboratory and outside the laboratory provide important adjuncts to safety and efficiency of operations but are insufficient alone to meet modern demands.

Persons who have gained the right of independent access to maximum-containment facilities are highly trained professionals who have earned the confidence of the laboratory director and are generally well respected and trusted by their colleagues (*11*). In addition, all such persons have had satisfactory background investigations and have obtained Department of Justice numbers in compliance with the Centers for Disease Control and Prevention Select Agent Program requirements. Furthermore, several BSL-4 laboratories are enacting some form of enhanced psychological screening and formal periodic monitoring of these persons. However, it is the responsibility of the laboratory director, the institutional leadership team, the safety and security staff, and the entire facility staff to ensure the personal safety of workers and to maintain the security of the facility and the infectious agents being studied.

## Conclusions

There is consensus among the directors of all major BSL-4 laboratories in the United States that a video monitoring system represents a more efficient, economical standard; provides greater assurance that pathogens are being properly manipulated; and offers an increased margin of employee safety and institutional security than does the 2-person security rule. Conversely, a requirement for 2 workers in the BSL-4 laboratory at all times may actually decrease compliance with the dual responsibilities of safety and security.
